# Supplementary figures and images for: Development of mammary cancer in γ-irradiated F1 hybrids of susceptible Sprague-Dawley and resistant Copenhagen rats, with copy-number losses that pinpoint potential tumor suppressors
Source: PLoS One. 2021 Aug 13;16(8):e0255968. doi: 10.1371/journal.pone.0255968 (PMC8362979; doi:10.1371/journal.pone.0255968)

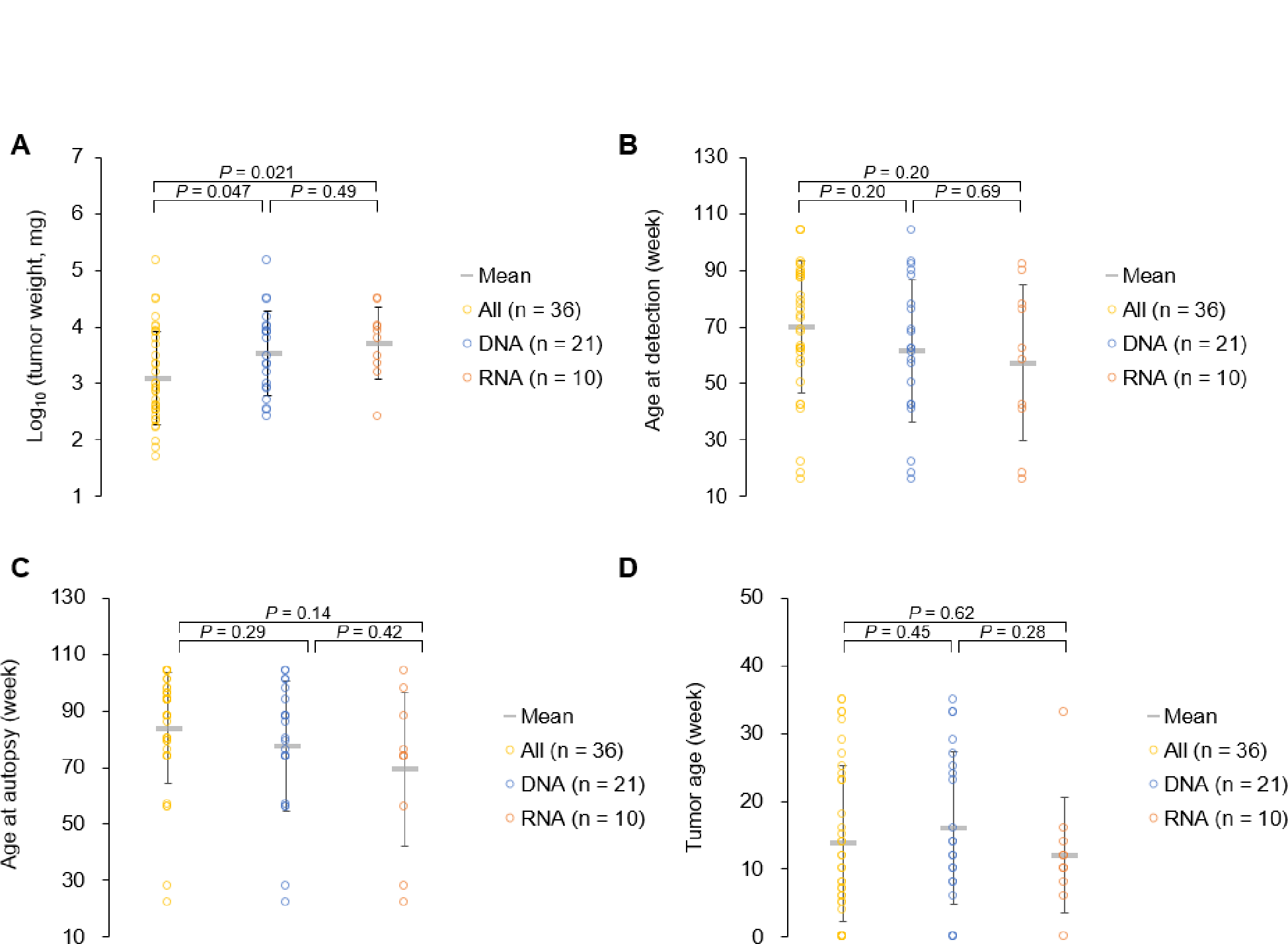

Supplement: S1 Fig — Distribution of tumor weight (A), age at tumor detection (B), age at autopsy (C), and tumor age (i.e., interval between tumor detection and autopsy) (D). Circles, individual tumors; horizontal and vertical bars, mean and SD. P values, Welch’s t test. (TIF) [file pone.0255968.s002.tif]

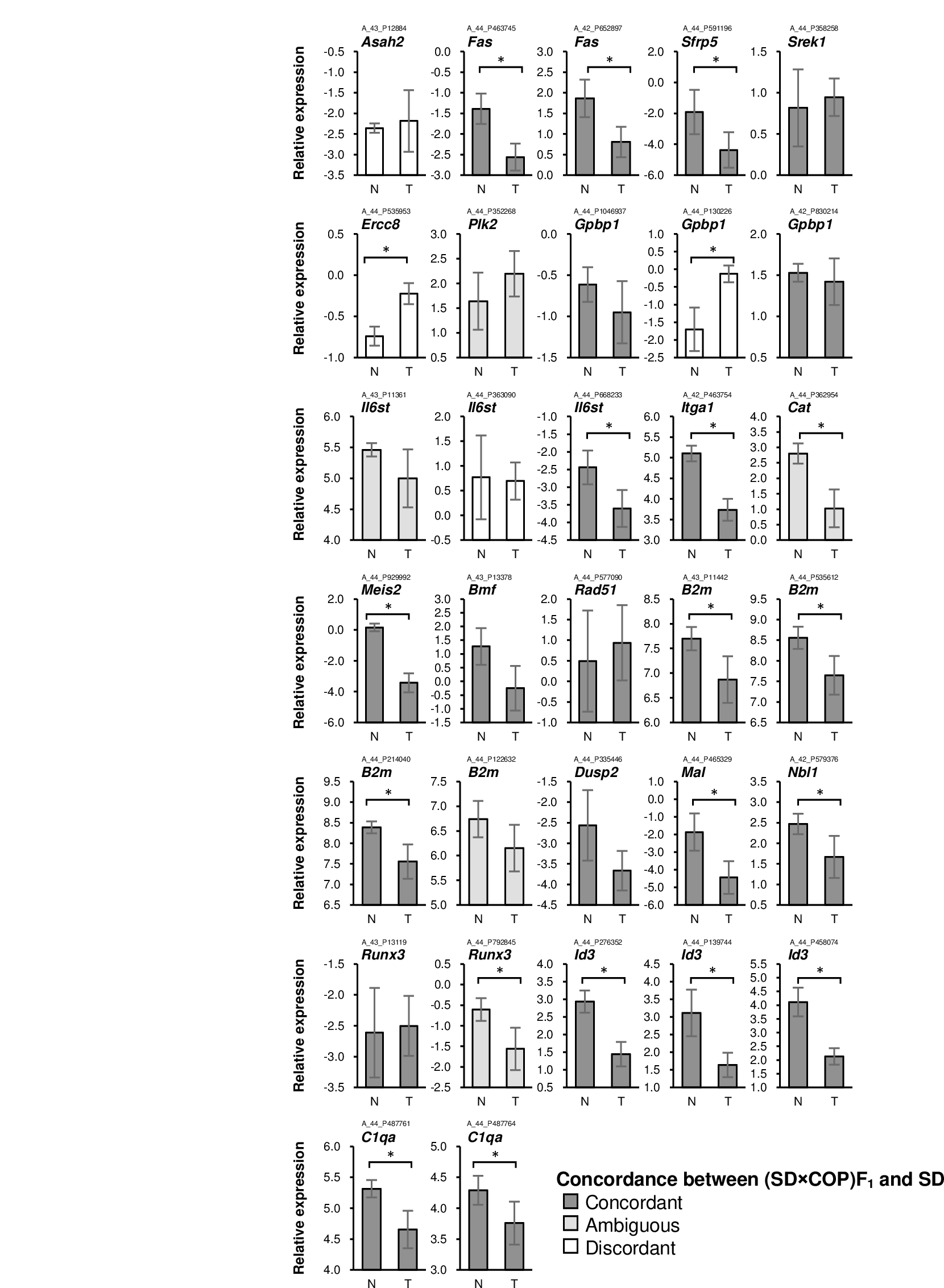

Supplement: S2 Fig — Genes in Fig 4 in mammary carcinomas from SD rats from a previous microarray analysis [24]. Expression levels are standardized against the 75th percentiles of all genes on individual microarrays and are expressed as log2 values. Probe IDs are shown above the gene symbols. Data are presented as the mean and SD. * P < 0.05, Mann-Whitney U test. (TIF) [file pone.0255968.s003.tif]
