# Supplementary material for: Development of mammary cancer in γ-irradiated F1 hybrids of susceptible Sprague-Dawley and resistant Copenhagen rats, with copy-number losses that pinpoint potential tumor suppressors
Source: PLoS One. 2021 Aug 13;16(8):e0255968. doi: 10.1371/journal.pone.0255968 (PMC8362979; doi:10.1371/journal.pone.0255968)
Supplement: Attachment — Submitted filename: Response letter.docx [file pone.0255968.s004.docx]

**I. RESPONSE TO REVIEWER #1**

We wish to express our appreciation to the reviewer for the insightful comments. Below, we have addressed the comments and have included the revisions made. Line numbers refer to those in the manuscript with track changes. Changes are indicated by the following rule.

1. Underlined: Added text

2. ~~Strikethrough~~: Deleted previous text

3. Red text: Changes relevant to the present comment

We have had all revised texts reviewed by two native English-speaking professionals at Bioedit (https://www.bioedit.com/).

**Overall remarks**

This manuscript described the genetic analysis of radiation-induced cancer induced in parental (SD and COP) as well as F1 rats. The use of susceptible and resistant rat strains in radiation carcinogenesis studies is not itself novel, and some features of this work have already been published, The molecular analysis of the genetic changes occurring in the F1 tumors has merit as an original observation. However, parts of the study are not clearly documented and there appears to be no consideration given to the fact that most, if not all, of the alterations may be due to genetic noise and/or can occur equally in sporadic mammary cancers.

The work comes from a laboratory internationally recognized for the quality of the animal research and all appropriate animal protection measures are followed. The paper is very clearly written and the language is excellent.

The results are of broad relevance in the fields of breast cancer and in [radiation-induced cancer, and therefore deserving of publication with some major and some minor changes needed to the manuscript (see below). Once these comments have been addressed or rebutted the manuscript is acceptable for publication.

**Response**: We appreciate the reviewer’s pertinent remarks on the present paper. Please see below for our point-by-point responses to each comment.

**Major comment I**

I) Variability in genetic purity, animal health, housing and treatment are all recognized as confounding factors in lifespan studies. Although the authors clearly state the study is based on historical data, it is not clear to the reviewer when the study of the different animals was done. Were the SD, COP and F1 rats all irradiated and observed at the same time, or were the studies done sequentially?

**Response**: Rats of the three strains were irradiated sequentially using the same irradiator and then observed in the same room for overlapping periods of time (Table R1).

**Table R1.** Period of experiment

| **Strain** | **Birth** | **Irradiation** | **Observation** |
| --- | --- | --- | --- |
| SD | Oct 2006–Jan 2007 | Nov 2006–Mar 2007  (in 6 batches) | Nov 2006–Sep 2008 |
| F_1_ | Mar 2007 | May 2007 (in 3 batches) | May 2007–Jun 2009 |
| COP | May 2007–Aug 2008 | Jul 2007–Oct 2008  (in 5 batches) | Jul 2007–Sep 2010 |

SD, Sprague-Dawley; COP, Copenhagen; F_1_, (SD×COP)F_1_.

To clarify this point, we have modified the description in the Materials and Methods as follows:

Lines 138–142: “Rats of the SD, (SD×COP)F_1_, and COP strains, born between October 2006 and August 2008, were ~~used in the study~~ sequentially irradiated (November 2006–March 2007, May 2007, and July 2007–October 2008, respectively) and observed for overlapping periods of time from November 2006 to September 2010.”

**Major comment II**

II) I was not able to understand the claims made for the relevance of the study results to radiation-induced versus sporadic cancer. I was unable to determine the sporadic cancer incidence in the three animal groups, nor was it possible to determine which genetic events are present in sporadic versus radiation-induced cancers.

**Response**: As we did not include non-irradiated groups in the study, we have carefully removed any claims implying radiation induction.

Running title: ~~Genomics of radiation-induced rat m~~ Mammary cancer in hybrid rats of susceptible and resistant strains after radiation exposure

Lines 1–3 (Title): “~~Radiation induces~~ Development of mammary cancer in γ-irradiated F_1_ hybrids of susceptible Sprague-Dawley and resistant Copenhagen rats, with copy number losses that pinpoint potential tumor suppressors”

Lines 33–40 (Abstract): “Copenhagen rats are highly resistant to ~~spontaneous~~ mammary carcinogenesis, even after treatment with ~~chemically induced,~~ chemical carcinogens and hormones ~~hormonally induced mammary cancers and,~~; most studies indicate that this is a dominant genetic trait. To test whether this trait is also dominant after ~~regarding~~ radiation exposure~~-induced carcinogenesis~~, we characterized the susceptibility of irradiated Copenhagen rats to ~~radiation-induced~~ mammary carcinogenesis, as well as its inheritance, and ~~to~~ identif~~y~~ied tumor-suppressor genes that, when inactivated or mutated, may contribute to carcinogenesis.”

Lines 55–58 (Abstract): “Some of the chromosomal losses identified have not been reported previously in chemically induced models, implying a novel mechanism inherent to ~~radiation-induced carcinogenesis~~ the irradiated model.”

Lines 116–118 (Introduction): “Thus, hybrid ~~animal~~s between strains with different tumor susceptibility offer ideal experimental tools for identifying potentially causative mutations.”

Lines 435–440 (Discussion): “We previously reported aberrations of genes related to the phosphoinositide 3-kinase (PI3K) pathway, including downregulation of *Pten* (1q52) and mutations of *Pik3r1* (2q12), in ~~radiation-induced rat~~ mammary cancers from ~~of~~ the same cohort of irradiated (SD×COP)F_1_ rats ~~cohort~~ [22], but the frequency of these mutations was low (i.e., 1 of 14 for each mutation).”

Lines 444–447 (Discussion): “By contrast, in our data, the polymorphic marker D1Mgh29, which is located on chromosome 1q52–54, shows a higher rate of loss of the potential susceptibility SD allele (2 of 3), suggesting a different role for this allele in the present model ~~radiation-induced carcinogenesis~~.”

Lines 458–460 (Discussion): “No ~~reported~~ QTL ~~exists~~ has been reported on chromosome 3q31–42, suggesting the existence of important unidentified determinants of susceptibility ~~to radiation-induced mammary carcinogenesis~~.”

Lines 519–524 (Discussion): “Taken together, the present study clearly indicates that irradiated (SD×COP)F_1_ hybrid rats are intermediately susceptible to ~~radiation-induced~~ mammary carcinogenesis, and hence~~,~~ are a useful model for exploring potentially causative gene mutations in ~~radiation-induced~~ mammary cancer. Chromosome regions 1q52–54, 2q12–15, and 3q31–42 and chromosome 5 are expected to harbor driver mutations relevant to ~~radiation-induced~~ mammary carcinogenesis.”

We have also added a sentence pointing to the need for future study on the attributability of the present findings to radiation:

Lines 506–508 (Discussion): “The present study has the following limitations. First, results should be compared between irradiated and non-irradiated groups to determine extent to which the present findings can be attributed to radiation exposure or genetic characteristics.”

**Major comment III**

III) The KM plots in Figure 1 do not show the claimed similarity between the incidence of MaCa in F1 and SD animals. The plot appears to this reviewer to show an intermediate phenotype suggestive of multiple alleles influencing susceptibility. Please either show statistical evidence or revise the text to remove suggestions that imply that the SD genotype has a dominant suppressive effect on radiation sensitivity.

**Response:** As the difference between the SD and F_1_ strains was close to statistical significance (log rank test *P* value = 0.1; hazard ratio = 0.46 [95% confidence interval, 0.21–1.0]) (Table 4), we have carefully removed any expression that implies similarity between cancer incidence in these strains.

Lines 46–48 (Abstract): “Unexpectedly, this resistance trait did not show dominant inheritance in F_1_ rats ~~but~~; rather, they exhibited intermediate susceptibility levels (i.e., between those of their parent strains) ~~similar to that of Sprague-Dawley rats~~.”

Lines 247–252 (Results): “Analysis of the time to first palpable mammary carcinoma indicated that COP rats developed carcinoma significantly less frequently than SD and (SD×COP)F_1_ rats, whereas the difference between SD and (SD×COP)F_1_ rats was also substantial, albeit with only marginal statistical significance, suggesting intermediate susceptibility of (SD×COP)F_1_ rats (Fig 1A, Table ~~3~~4).”

Lines 395–398 (Discussion): “We examined the mammary cancer susceptibility of irradiated (SD×COP)F_1_ hybrids of susceptible SD and resistant COP rats and found that they had intermediate ~~retain the relatively~~ susceptibility levels ~~of the SD strain and~~; thus, they are useful for exploring cancer-causing gene mutations.”

Lines 425–428 (Discussion): “This is in stark contrast to our finding that the susceptibility of (SD×COP)F_1_ hybrids was intermediate, i.e., between ~~close to~~ that of ~~the SD~~ its parent strains,~~. However,~~ which is logical, as this trait is known to be polygenic [15]~~, it makes sense that the susceptibility of (SD×COP)F~~_~~1~~_ ~~hybrids was rather high in the present study~~.”

Lines 519–522 (Discussion): “Taken together, the present study clearly indicates that irradiated (SD×COP)F_1_ hybrid rats are intermediately susceptible to ~~radiation-induced~~ mammary carcinogenesis, and hence are~~,~~ a useful model for exploring potentially causative gene mutations in ~~radiation-induced~~ mammary cancer.”

Please note that we have kept the following sentence as it appears in the previous manuscript draft; this is because some of the data presented in Table 3 indicate similarity between the two strains.

Lines 246–247 (Results): “(SD×COP)F_1_ rats showed susceptibility that was close to that of SD rats (Table ~~2~~3).”

**Major comment IV**

IV) The assignment of causality to genetic alterations found in a tumor can be misleading. In the final sentence of the introduction the authors imply the changes they observe may be causal. This is not a valid argument, rather the changes shown can only be termed as being associated with the development of the cancer. If they are passengers, causal or enabling mutations remains unproven.

**Response:** We have modified the last sentence of the Introduction, as well as a sentence in the Discussion, as suggested.

Lines 124–128 (Introduction): “Taking advantage of the mammary carcinomas developed in the (SD×COP)F_1_ rats, we then located chromosomal regions exhibiting aberrations with the aim of identifying candidate tumor-suppressor genes ~~that~~ associated with~~, when inactivated or mutated, may be causative for~~ carcinogenesis.”

Lines 519–522 (Discussion): “Taken together, the present study clearly indicates that irradiated (SD×COP)F_1_ hybrid rats are intermediately susceptible to ~~radiation-induced~~ mammary carcinogenesis, and hence~~,~~ are a useful model for exploring potentially causative gene mutations in ~~radiation-induced~~ mammary cancer.”

**Major comment V**

V) The possible strain-specific differences in tumor type (Adenocarcinoma, fibroadenoma or luminal/non-luminal) and location have not been discussed. The histopathological analysis of the tumors is missing. (See also point iii).

**Response:** We analyzed possible strain differences in tumor type (adenocarcinoma, fibroadenoma or adenoma) and location (abdomino-inguinal or thoracic).

First, the analysis of tumor type did not support any difference in the malignant-to-benign ratio, but did support more frequent presentation of adenoma as benign tumors in COP rats rather than in SD and F_1_ rats (see Table R2 below).

**Table R2. Tumor type**

| **Strain** | **1) All tumors** |  |  | **2) Benign tumors** |  |
| --- | --- | --- | --- | --- | --- |
|  | Malignant *^a^* | Benign *^b^* |  | Fibroadenoma | Adenoma |
| SD | 18/71 (25%) | 53/71 (75%) |  | 53/53 (100%) | 0/53 (0%)*** |
| COP | 9/20 (45%) | 11/20 (55%) |  | 7/11 (64%) | 4/11 (36%) |
| F_1_ | 36/104 (35%) | 68/104 (65%) |  | 65/68 (96%) | 3/68 (4%)** |

*^a^* Carcinoma. *^b^* Fibroadenoma and adenoma combined. ***P* < 0.01, ****P* < 0.001 *vs.* COP.

SD, Sprague-Dawley; COP, Copenhagen; F_1_, (SD×COP)F_1_.

Regarding tumor location, carcinomas tended to develop in the abdomino-inguinal mammary gland chains, and this did not differ significantly among strains. Benign mammary tumors developed equally in the abdomino-inguinal and thoracic glands, again with no significant difference among strains (see Table R3 below).

**Table R3. Tumor location**

| **Strain** | **Location (carcinoma)** | |  | **Location (benign)** | |
| --- | --- | --- | --- | --- | --- |
|  | Abdomino-inguinal | Thoracic |  | Abdomino-inguinal | Thoracic |
| SD | 12 (67%) | 6 (33%) |  | 27 (51%) | 26 (49%) |
| COP | 8 (89%) | 1 (11%) |  | 5 (45%) | 6 (55%) |
| F_1_ | 18 (50%) | 18 (50%) |  | 38 (56%) | 30 (44%) |

SD, Sprague-Dawley; COP, Copenhagen; F_1_, (SD×COP)F_1_.

We have included these data in Table 2, and in the text of the Results section, as follows:

Lines 235–239 (Results): “All carcinomas and benign tumors were palpable, and no additional tumors in these categories were discovered upon necropsy. Neither the location of tumors (abdomino-inguinal or thoracic) nor the malignant-to-benign ratio differed among strains, although COP rats had more adenomas than SD and (SD×COP)F_1_ rats as benign tumors (Table 2).”

We did not analyze tumor subtype (luminal, non-luminal, etc.) in the present study; therefore, we have summarized previous studies and discussed the potential importance of tumor subtype as follows:

Lines 506–509 (Discussion): “The present study includes the following limitations. … Third, it is unclear whether the mammary carcinoma subtype is strain-dependent. Our previous studies on mammary carcinomas in (SD×COP)F_1_ (*n* = 24 tumors) and SD (*n* = 85 tumors) rats indicated that they are mainly (70%–90%) of the luminal subtype [22, 32, 33], consistent with reports from other laboratories [47, 48]. Further studies are required to clarify whether this applies to COP rats, as the number of tumors obtained in our study was small (*n* = 9) due to the high resistance of the strain.”

**Major comment VI**

VI) The different sensitivities of the two strains, and the F1, are probably rather specific to the dose of radiation and age at irradiation, with each strain having different tumor growth rates that influence the time of detection. The factors governing the decision to use a single dose of 4Gy and the selection for the age at irradiation should therefore be explained (I assume coherence with previous literature studies?).

**Response:** We chose to use the current radiation dose (4 Gy), dose rate (0.5 Gy/min), and age at irradiation (7 weeks) based on previous studies. These conditions have been proven to induce significant mammary carcinogenesis in SD rats. Mammary carcinogenesis exhibits a dose-dependent increase in SD rats up to at least 4 Gy; lowering the radiation dose rate significantly reduces the incidence of both carcinoma and benign tumors (ref. 17). Our previous experiment indicated that irradiation of 3-week-old rats with 2–4 Gy results in attenuated mammary carcinogenesis, compared with 7-week-old rats (refs. 23 and 24). Thus, we have added a sentence about the choice of exposure conditions. Furthermore, because the conditions of radiation exposure can vary and are a matter of intense investigation, we have added a brief discussion of this point to the revised manuscript.

Lines 141–145 (Materials and Methods): “Briefly, 7-week-old female rats were whole-body γ-irradiated (^137^Cs, 4 Gy, 0.5 Gy/min) and then palpated weekly for the remainder of their lifetime to detect tumors. Based on data from our previous studies [17, 23, 24], the dose and dose rate of radiation, and the age at irradiation, were selected as conditions that induced maximal mammary carcinogenesis.”

Lines 497–503 (Discussion): “The present study has the following limitations. ... Second, our results may have been affected by variation in exposure conditions, such as radiation dose, dose rate, fractionation regimen, radiation type, age at time of exposure, hormonal conditions at time of exposure, and whether the whole or partial body was irradiated. This point remains open for future study.”

**Major comment VII**

VII) Gene expression in normal tissue is used as control for expression in cancers. This was done on a subset of tumors. How were these tumors selected and what type of tumors were considered? The influence of size and location, as well as tumor type should be considered. Moreover, the use of normal mammary tissue includes of course considerable cell heterogeneity, so it is important to describe how normal tissue was selected and prepared.

**Response: 1) Selection of tumors.** As tumors of a certain volume were required for pathology, sometimes molecular analyses could not be conducted for small tumors. Further, we prioritized DNA analysis in our molecular studies; therefore, frozen samples were only stored for RNA extraction if there was sufficient volume remaining. Consequently, the size distribution of tumors used for DNA (*n* = 21) and RNA (*n* = 10) analyses was biased upwards compared with the entire population of tumors (*n* = 36), while tumors used for DNA and RNA analyses were of similar size (see Figure R1A below). Of the 21 tumors used for DNA, 11 were not available for RNA because of their small size or autolysis. Nevertheless, tumor location, age at detection, age at collection (i.e., age at autopsy), and tumor age (i.e., interval between tumor detection and collection), did not differ significantly among tumors used for DNA and RNA analysis and all tumors collected (Figure R1B–D, Table R4). We have included these data in Supporting Information S2 in the revised manuscript. We have also modified the text as follows:

Lines 147–149 (Materials and Methods): “~~The a~~Animals that showed signs of general deterioration (including signs of natural death) were euthanized by exsanguination under isoflurane anesthesia and autopsied; animals found dead were also autopsied.”

Lines 156–157 (Materials and Methods): “~~Mammary~~ Remaining portions of mammary carcinomas and normal glands were frozen in liquid nitrogen and stored at −80°C.”

Lines 163–168 (Materials and Methods): “DNA extraction from all frozen tumors that were diagnosed as carcinomas (*n* = 21) was prioritized; of these 21 tumors, those that were freshly collected and had an available remaining portion (*n* = 10) were used for RNA extraction. This selection of tumors tended to be larger, but were not biased in terms of location, age at detection, age at collection, or time interval between detection and collection, relative to the entire set of carcinomas (*n* = 36) (see Supporting Information S2). Genomic DNA and total RNA were extracted …”

Lines 734–737 (Supporting Information): “**S2 Figure. Effect of selection of tumors.** Distribution of tumor weight (A), age at tumor detection (B), age at autopsy (C), and tumor age (i.e., interval between tumor detection and autopsy) (D). Circles, individual tumors; horizontal and vertical bars, mean and SD. *P* values, Welch’s *t* test.”

**Figure R1. Effect of tumor selection.** Distribution of tumor weight (A), age at tumor detection (B), age at autopsy (C), and tumor age (i.e., interval between tumor detection and autopsy) (D). Circles, individual tumors; horizontal and vertical bars, mean and SD. *P* values, Welch’s *t* test.

**Table R4.** Locations of all tumors and those used in DNA and RNA analyses

| **Location** | **All (*n* = 36)** | **DNA (*n* = 21)** | **RNA (*n* = 10)** |
| --- | --- | --- | --- |
| Abdomino-inguinal | 18 (50%) | 10 (48%) | 5 (50%) |
| Thoracic | 18 (50%) | 11 (52%) | 5 (50%) |

**2) Preparation of normal tissue samples.** As mammary epithelium is embedded in fat tissue and not necessarily macroscopically identifiable, collection of tissue distant from the nipple may result in retrieval of stromal tissues, which do not contain epithelium. Therefore, normal mammary tissue samples were collected from an approximately 1 cm^2^ region proximal to the abdominal nipple to ensure that epithelium was included in the samples. We also took care to avoid collection of lymph nodes, skin, and muscle. We have added this information to the Materials and Methods, as follows:

Lines 153–157 (Materials and Methods): “The palpation record was used to determine the age at which tumors first developed. Normal mammary tissue was collected from an approximately 1 cm^2^ region proximal to an abdominal nipple to ensure inclusion of mammary epithelium; special care was taken to exclude lymph nodes, skin, and muscle.”

**Major comment VIII**

VIII) The genetic findings in the recent study of Moriyama, including the senior author of this paper using N- irradiated SD rats (Moriyama 2021 DOI: https://doi.org/10.21873/anticanres.14751) should be compared in much greater detail to indicate differences.

**Response:** We examined DNA copy number changes of breast cancer–relevant genes in the tumors analyzed in this study, as well as those reported by Moriyama et al. (Table R5). The major findings were:

1) There was a higher frequency of copy number changes in the present study than that reported by Moriyama et al.

2) The two studies had a concordant higher frequency of losses than gains.

3) Multiple tumors had loss of one of *Pten*, *Cdkn2a*, or *Cdkn2b*.

We have added this information to Table 6, with some discussion, in the revised manuscript.

**Table R5.** Frequency of tumors with meaningful copy number changes in breast cancer–relevant genes, compared with that reported by Moriyama et al. (2021) (*shaded cells indicate non-zero results)*

| **Gene symbol** | **Present study** | | **Moriyama et al. (2021)** | | | | | |
| --- | --- | --- | --- | --- | --- | --- | --- | --- |
|  | γ rays (4 Gy), (SD×COP)F_1_ (*n* = 21) | | γ rays (4 Gy),  SD (*n* = 10) | | Neutrons (1 Gy), SD (*n* = 8) | | Non-irradiated, SD (*n* = 10) | |
|  | Loss | Gain | Loss | Gain | Loss | Gain | Loss | Gain |
| *Esr1* | 2 (10%) | 0 (0%) | 0 (0%) | 0 (0%) | 0 (0%) | 0 (0%) | 0 (0%) | 0 (0%) |
| *Cnot3* | 2 (10%) | 0 (0%) | 0 (0%) | 0 (0%) | 0 (0%) | 0 (0%) | 0 (0%) | 0 (0%) |
| *Cic* | 2 (10%) | 0 (0%) | 0 (0%) | 0 (0%) | 0 (0%) | 0 (0%) | 0 (0%) | 1 (10%) |
| *Palb2* | 3 (14%) | 0 (0%) | 0 (0%) | 0 (0%) | 0 (0%) | 0 (0%) | 0 (0%) | 0 (0%) |
| *Men1* | 2 (10%) | 0 (0%) | 0 (0%) | 0 (0%) | 0 (0%) | 0 (0%) | 0 (0%) | 0 (0%) |
| *Pten* | 5 (24%) | 0 (0%) | 2 (20%) | 0 (0%) | 0 (0%) | 0 (0%) | 0 (0%) | 0 (0%) |
| *Pik3r1* | 7 (33%) | 0 (0%) | 0 (0%) | 0 (0%) | 0 (0%) | 0 (0%) | 0 (0%) | 0 (0%) |
| *Map3k1* | 7 (33%) | 0 (0%) | 0 (0%) | 0 (0%) | 0 (0%) | 0 (0%) | 0 (0%) | 0 (0%) |
| *Fbxw7* | 3 (14%) | 0 (0%) | 0 (0%) | 0 (0%) | 0 (0%) | 0 (0%) | 0 (0%) | 0 (0%) |
| *Notch2* | 3 (14%) | 0 (0%) | 0 (0%) | 0 (0%) | 0 (0%) | 0 (0%) | 0 (0%) | 0 (0%) |
| *Notch1* | 1 (5%) | 0 (0%) | 0 (0%) | 0 (0%) | 0 (0%) | 0 (0%) | 0 (0%) | 1 (10%) |
| *Bub1b* | 5 (24%) | 0 (0%) | 0 (0%) | 0 (0%) | 0 (0%) | 0 (0%) | 0 (0%) | 0 (0%) |
| *Foxp1* | 1 (5%) | 0 (0%) | 0 (0%) | 0 (0%) | 0 (0%) | 0 (0%) | 0 (0%) | 0 (0%) |
| *Cdkn1b* | 1 (5%) | 0 (0%) | 0 (0%) | 0 (0%) | 0 (0%) | 0 (0%) | 0 (0%) | 0 (0%) |
| *Cdkn2a* | 8 (38%) | 0 (0%) | 1 (10%) | 0 (0%) | 2 (25%) | 0 (0%) | 0 (0%) | 0 (0%) |
| *Cdkn2b* | 8 (38%) | 0 (0%) | 1 (10%) | 0 (0%) | 2 (25%) | 0 (0%) | 0 (0%) | 0 (0%) |
| *Arid1a* | 7 (33%) | 0 (0%) | 0 (0%) | 0 (0%) | 0 (0%) | 0 (0%) | 0 (0%) | 0 (0%) |
| *Spen* | 7 (33%) | 0 (0%) | 0 (0%) | 0 (0%) | 0 (0%) | 0 (0%) | 0 (0%) | 0 (0%) |
| *Msh2* | 2 (10%) | 0 (0%) | 0 (0%) | 0 (0%) | 0 (0%) | 0 (0%) | 1 (10%) | 0 (0%) |
| *Dnmt3a* | 2 (10%) | 0 (0%) | 0 (0%) | 0 (0%) | 0 (0%) | 0 (0%) | 0 (0%) | 0 (0%) |
| *Smarca4* | 2 (10%) | 0 (0%) | 0 (0%) | 0 (0%) | 0 (0%) | 0 (0%) | 0 (0%) | 0 (0%) |
| *Atm* | 2 (10%) | 0 (0%) | 0 (0%) | 0 (0%) | 0 (0%) | 0 (0%) | 0 (0%) | 0 (0%) |
| *Setd2* | 2 (10%) | 0 (0%) | 0 (0%) | 0 (0%) | 0 (0%) | 0 (0%) | 0 (0%) | 0 (0%) |
| *Mlh1* | 2 (10%) | 0 (0%) | 0 (0%) | 0 (0%) | 0 (0%) | 0 (0%) | 1 (10%) | 0 (0%) |
| *Casp8* | 2 (10%) | 0 (0%) | 0 (0%) | 0 (0%) | 0 (0%) | 0 (0%) | 1 (10%) | 0 (0%) |
| *Crebbp* | 0 (0%) | 1 (5%) | 0 (0%) | 0 (0%) | 0 (0%) | 0 (0%) | 0 (0%) | 0 (0%) |
| *Axin1* | 1 (5%) | 1 (5%) | 0 (0%) | 0 (0%) | 0 (0%) | 0 (0%) | 0 (0%) | 0 (0%) |
| *Ncor1* | 1 (5%) | 1 (5%) | 0 (0%) | 0 (0%) | 0 (0%) | 0 (0%) | 0 (0%) | 0 (0%) |
| *Map2k4* | 1 (5%) | 1 (5%) | 0 (0%) | 0 (0%) | 0 (0%) | 0 (0%) | 0 (0%) | 0 (0%) |
| *Tp53* | 1 (5%) | 1 (5%) | 0 (0%) | 0 (0%) | 0 (0%) | 0 (0%) | 0 (0%) | 0 (0%) |
| *Nf1* | 1 (5%) | 1 (5%) | 0 (0%) | 0 (0%) | 0 (0%) | 0 (0%) | 0 (0%) | 0 (0%) |
| *Erbb2* | 0 (0%) | 1 (5%) | 0 (0%) | 0 (0%) | 0 (0%) | 0 (0%) | 0 (0%) | 0 (0%) |
| *Cux1* | 0 (0%) | 1 (5%) | 0 (0%) | 0 (0%) | 0 (0%) | 0 (0%) | 0 (0%) | 0 (0%) |
| *Tbx3* | 0 (0%) | 1 (5%) | 0 (0%) | 0 (0%) | 0 (0%) | 0 (0%) | 0 (0%) | 0 (0%) |
| *Nf2* | 1 (5%) | 0 (0%) | 0 (0%) | 0 (0%) | 0 (0%) | 0 (0%) | 0 (0%) | 0 (0%) |
| *Rb1* | 1 (5%) | 0 (0%) | 1 (10%) | 0 (0%) | 0 (0%) | 0 (0%) | 0 (0%) | 0 (0%) |
| *Znf703* | 0 (0%) | 1 (5%) | 0 (0%) | 0 (0%) | 0 (0%) | 0 (0%) | 0 (0%) | 0 (0%) |
| *Fgfr1* | 0 (0%) | 2 (10%) | 0 (0%) | 0 (0%) | 0 (0%) | 0 (0%) | 0 (0%) | 0 (0%) |
| *Gata3* | 3 (14%) | 2 (10%) | 0 (0%) | 0 (0%) | 0 (0%) | 0 (0%) | 0 (0%) | 0 (0%) |
| *Apc* | 3 (14%) | 0 (0%) | 0 (0%) | 0 (0%) | 0 (0%) | 0 (0%) | 0 (0%) | 0 (0%) |
| *Smad4* | 3 (14%) | 0 (0%) | 0 (0%) | 0 (0%) | 0 (0%) | 0 (0%) | 1 (10%) | 0 (0%) |
| *Prdm1* | 3 (14%) | 0 (0%) | 0 (0%) | 0 (0%) | 0 (0%) | 0 (0%) | 0 (0%) | 0 (0%) |
| *Bcor* | 4 (19%) | 0 (0%) | 0 (0%) | 0 (0%) | 0 (0%) | 0 (0%) | 1 (10%) | 0 (0%) |
| *Atrx* | 4 (19%) | 0 (0%) | 0 (0%) | 0 (0%) | 0 (0%) | 0 (0%) | 1 (10%) | 0 (0%) |
| *Stag2* | 4 (19%) | 0 (0%) | 0 (0%) | 0 (0%) | 0 (0%) | 0 (0%) | 1 (10%) | 0 (0%) |

Lines 294–302 (Results): “We identified many genes relevant to human breast cancer [27] in the chromosomal regions exhibiting copy number changes (Table 46). Interestingly, the number of genes affected by copy number loss was positively correlated with age at detection (Fig. 2B, *P* < 0.01), which could be explained by the correlation with the number of chromosomes with large deletions spanning > 80% of the chromosome (Fig. 2C, *P* < 0.01). The frequencies of copy number losses and gains involving human-relevant tumor suppressors and proto-oncogenes, respectively, were much higher than those reported in a previous study on SD rats [33] (Table 6, two rightmost columns).”

Lines 402–405 (Discussion): “This frequency is higher than the AI frequency reported in radiation-induced mammary cancer of (WF×COP)F_1_ rats (4–13%) [18], and higher than the frequency of copy number changes in breast cancer–relevant genes in SD rats (4–11%) [33].”

Lines 440–441 (Discussion): “Copy number loss of *Pten* observed in (SD×COP)F_1_ rats (24%) has also been reported in SD rats [33].”

**Major comment IX**

IX) The tumor incidence data is censored, but it is not clear what exclusion criteria were used, nor if the F1 and COP had different competing causes.

**Response:** We censored animals subjected to euthanasia for humane reasons, as well as animals that died naturally. This has been added to the Materials and Methods.

Lines 147–150 (Materials and Methods): “~~The a~~Animals that showed signs of general deterioration (including signs of natural death) were euthanized by exsanguination under isoflurane anesthesia and autopsied; animals found dead were also autopsied. Animals terminated before tumor development were censored.”

The causes of termination are summarized in Table 5 in the revised manuscript (please see below). Fisher’s exact test indicated no significant difference among strains. This result has been added to the Results section. Relevant raw data have been added to Supporting Information S1 (Sheet 1).

Lines 251–253 (Results): “…, suggesting intermediate susceptibility of the (SD×COP)F_1_ rats (Fig 1A, Table ~~3~~4). Susceptibility of (SD×COP)F_1_ rats to benign mammary tumors was intermediate between the parental strains (Fig. 1B, Table ~~3~~4). Causes of censoring did not differ among strains (Table 5).”

**Table 5. Causes of censoring** (note that the table number is that used in the revised manuscript)

| **Analysis** | **Cause** | **Strain*** |  |  |
| --- | --- | --- | --- | --- |
|  |  | SD | COP | (SD×COP)F_1_ |
| Carcinoma | Mammary neoplasm | 4 | 2 | 5 |
|  | Other neoplasms | 1 | 3 | 3 |
|  | Non-neoplasms | 0 | 3 | 0 |
|  | Unidentified | 2 | 4 | 2 |
| Benign tumors | Mammary neoplasm | 2 | 1 | 5 |
|  | Other neoplasms | 1 | 2 | 1 |
|  | Non-neoplasms | 0 | 1 | 0 |
|  | Unidentified | 0 | 5 | 2 |

*There was no significant difference among strains (Fisher’s exact test). SD, Sprague-Dawley; COP, Copenhagen.

**Major comment X**

X) The very important data presented in Figure 4 is drawn from a subset of the tumors available. How was the selection made and most importantly how was the quantification of expression mathematically performed. A comment on the change of expression of these genes seen in COP or SD tumors would greatly improve the manuscript.

**Response:** Information on how the selection was made has been added to the revised manuscript in response to comment VII. Calculation of the expression values and a relevant citation have been added to the Materials and Methods.

Lines 217–219 (Materials and Methods): “The expression levels of target genes were normalized to those ~~that~~ of *Gapdh* and expressed relative to the value of an arbitrarily selected normal tissue sample using the 2^–ΔΔCT^ method [30].”

Lines 649–651 (Reference): “30. Livak KJ, Schmittgen TD. Analysis of relative gene expression data using real-time quantitative PCR and the 2^–ΔΔCT^ method. Methods. 2001;25(4):402-8.” *(citation numbers have been shifted accordingly)*

We did not measure gene expression levels in carcinomas from SD or COP rats in the present study. Therefore, we assessed our previous data from a gene expression microarray experiment (ref. 24 in the revised manuscript; deposited in the Gene Expression Omnibus database, GSE22770) on mammary carcinomas from SD rats (irradiated at 2 Gy of ^137^Cs γ rays at 7 weeks-of-age) and compared them with our current data from the (SD×COP)F_1_ strain (Figure R2 below). This comparison indicated the following:

i) Many genes showed concordant significant downregulation (*Fas*, *Sfrp5*, *Itga1*, *Meis2*, *B2m*, *Mal*, *Nbl1*, *Id3*, *C1qa*) or concordant absence of significant change (*Srek*, *Bmf*, *Rad51*, *Dusp2*) between the strains (dark bars in Figure R2).
ii) Some genes exhibited significant changes in tumors of one strain that were discordant in the other strain (*Asah2*, *Ercc8*, *Gpbp1*; white bars in Figure R2).
iii) Results were ambiguous regarding other genes due to concordant trends in change, which did not reach statistical significance in either strain (*Plk2*, *Il6st*, *Cat*, *Runx3*; light bars in Figure R2), or due to expression levels below the detection limit (*Ifit1*, *Cenpk*, *Trp53bp1*, *Bcl2l11*; not shown in Figure R2).

**Figure R2. Expression of genes in SD rat mammary carcinomas.** Genes referred to in Figure 4 of the manuscript in mammary carcinomas of SD rats in a previous microarray analysis. Expression levels are standardized against the 75th percentiles of all genes on individual microarrays and are expressed as log_2_ values. Probe IDs are shown above the gene symbols. Data are presented as the mean and SD. * *P* < 0.05, Mann-Whitney *U* test.

Thus, comparison of the data presented in Figure 4 from (SD×COP)F_1_ rats with that from our previous microarray data from SD rats supported common downregulation of *Fas*, *Sfrp5* (chromosome 1q52–54), *Itga1* (chromosome 2q12–15), *Meis2*, *B2m*, *Mal*, *Nbl1* (chromosome 3q31–42), *Id3*, and *C1qa* (chromosome 5) in mammary carcinomas, and hence their role in tumor suppression. We have added this to Discussion.

Lines 460–468 (Discussion): “… A further search for candidate causative genes in this region is thus warranted, and downregulated genes identified in the current study, namely *Meis2*, *B2m*, *Mal*, and *Nbl1*, are ~~among such~~ candidates for further study. Comparison with previous measurements of gene expression changes in radiation-induced SD rat mammary carcinoma [24] (see Supporting Information S3) supports downregulation of *Fas*, *Sfrp5* (chromosome 1q52–54), *Itga1* (chromosome 2q12–15), *Meis2*, *B2m*, *Mal* (chromosome 3q31–42), *Id3*, *C1qa, and Nbl1* (chromosome 5), reinforcing the roles of these genes as potential tumor suppressors. A previous study on irradiated (WF×COP)F_1_ rats…”

Lines 738–742 (Supporting Information): “**S3 Figure. Expression of genes in SD rat mammary carcinomas.** Genes in Figure 4 in mammary carcinomas from SD rats from a previous microarray analysis [24]. Expression levels are standardized against the 75^th^ percentiles of all genes on individual microarrays and are expressed as log_2_ values. Probe IDs are shown above the gene symbols. Data are presented as the mean and SD. * *P* < 0.05, Mann-Whitney *U* test.”

**Minor comment i**

i) Animal numbers used are hard to find in the present manuscript, it would greatly improve readability if the n= convention was used in the text.

**Response:** We have introduced the ‘*n* =’ convention for numbers of animals and tumors, as suggested (lines 163, 165, 168, 235–236, 285, 501, 514, and 517; Table 6).

**Minor comment ii**

ii) The data in Table 4 show human genes mapping to regions of CNV/ in the rat tumors needs clarification. How were these homologies mapped?

**Response:** We did not actually map the regions of the rat genome with CNVs to the human genome. In this study, we first focused our attention on a set of human genes (*n* = 94), which was previously reported to be repeatedly affected in breast cancer [27]. The rat orthologs of these 94 genes were then determined using the UCSC Genome Browser Database; the providers of the database explain that cross-species homology annotations were generated by alignments of genomic sequences with their repetitive elements masked [28]. After rat orthologs were determined, we simply looked for CNVs of the corresponding regions in the microarray data. As our original description of this method was somewhat confusing, we have made the following corrections:

Lines 187–189 (Materials and Methods): “~~Chromosomal regions corresponding to those~~ Rat orthologs of human genes relevant to ~~human~~ breast cancer [27] were ~~located~~ identified in the rn4 rat genome assembly using the UCSC Genome Browser (https://genome.ucsc.edu/) [28].”

Lines 640–643 (References): “28. Karolchik D, Baertsch R, Diekhans M, Furey TS, Hinrichs A, Lu YT, et al. The UCSC Genome Browser Database. Nucleic Acids Res. 2003;31(1):51-4. Epub 2003/01/10. doi: 10.1093/nar/gkg129. PubMed PMID: 12519945; PubMed Central PMCID: PMCPMC165576.”

**Minor comment iii**

iii) The paper would have been greatly improved by inclusion of discussion on alternative causes of the different susceptibilities e.g. mammary virus tumor burden, strain genetic contamination, animal weight and lifespan, speed of tumor growth in the two strains, tumor multiplicity.

**Response:** We are grateful for this insightful feedback. We have added the results of body weight analysis and new discussions. These discussions are supported by the evidence and analyses described in the subsequent text below.

Lines 254–257 (Results): “Throughout the course of the experiment, SD rats were significantly heavier than COP rats, while the weights of (SD×COP)F_1_ rats were intermediate (Fig 1C).”

Lines 271–274 (legend, Fig. 1): “A, carcinoma; B, benign tumors (fibroadenoma and adenoma). Data ~~for~~ from SD rats were ~~previously~~ reported previously [17] and reanalyzed. C, Body weight during the experiment (mean and standard deviation). ****P* < 0.001 between strains, two-way analysis of variance.”

Lines 478–494 (Discussion): “Beside the above-mentioned susceptibility loci, several factors should be considered that may have caused the observed strain difference. Burden of mammary tumor virus has been related to mammary tumor susceptibility in some mouse strains [40], and a counterpart tumor virus has been reported in rats [41], but has not been confirmed extensively; however, we cannot completely rule out the possibility that the strain difference observed in the current study is due to virus burden. Genetic contamination has been reported as another factor influencing disease susceptibility in specific rat strains [42, 43]. The current SD and COP strains were maintained under strict management; nevertheless, as genetic tests were not conducted, the possibility of contamination cannot be excluded. It is also possible that systemic factors affect tumor development. Long-term hormone administration to COP rats has been reported to promote development of mammary tumors that would otherwise undergo spontaneous regression [44]. Fat tissue is a source of estrogen, especially after senescence of ovarian function; thus, obesity and being overweight promote breast cancer development [45]. The differences in body weight among rat strains reported herein, which is concordant with that associated with mammary cancer susceptibility, is therefore a plausible factor that could explain the observed susceptibility.”

The above discussion is backed by the following lines of evidence and analyses.

1) *Mammary tumor virus*. It is established that murine mammary tumor virus (MMTV) burden greatly influences strain differences in mouse mammary tumor susceptibility (for example, ref. 40). Previous discussions have postulated that susceptibility of rats to mammary tumors could also be affected by mammary tumor virus (such as R-35 virus, ref. 41). This hypothesis has not been extensively addressed to date, and we do not have evidence regarding whether or not virus infection is relevant to the strain difference described here.

2) *Strain genetic contamination*. Genetic contamination has been reported in several rat strains (ref. 42 and 43). The Jcl:SD strain used in this study was maintained in a dedicated room by the breeder to prevent genetic contamination; however, no genetic tests were conducted (Clea Japan Inc., personal communication, May 2021). The COP/Hsd strain was introduced to the authors’ facility and maintained for 8–11 generations by brother-sister mating before use. Our mating records and maintenance of the characteristic coat color of the COP/Hsd strain (white with a brown hood) represent evidence against genetic contamination in our facility, but again, genetic tests have not been conducted. Thus, it is unlikely, but not impossible, that genetic contamination is a contributory factor in our findings.

3) *Animal body weight*. Comparison of animal body weights indicated clear differences among strains, with SD heavier than COP, and F1 animals an intermediate weight (Figure R3). Adipose tissue-derived estrogens have been implicated as a mechanism promoting breast cancer in women. Thus, multiple genetic factors governing body weight may have contributed to the strain difference observed in this study.

**Figure R3.** Body weight of SD, COP, and F1 rats. ****P* < 0.001 between strains, analysis of variance.

4) *Animal lifespan*. In general, long lifespan contributes to high cumulative incidence of cancer. Lifespan differed among strains in the present study (Table 3, age at autopsy). Cumulative incidence data (Table 3, rats with a tumor [%]), as well as Kaplan-Meier (Fig. 1) and Cox regression (Table 4) analyses, indicated that the strain difference was more prominent after adjustment for the lifespan difference. Thus, lifespan alone does not explain the observed strain difference.

5) *Tumor growth rate*. As the present study used palpation to detect tumors, the rate of tumor growth before their detection is a factor that may have influenced the difference in age at first tumor palpation among strains. It was not possible to estimate the tumor growth rate before they were detected. In addition, we did not measure tumor size in a chronological manner. Rather, we plotted tumor weight at autopsy against time since detection (i.e., the time between first palpation and autopsy), in an attempt to estimate the initial tumor growth rate after detection (see Figure R4 below). Our data indicate a trend of increased tumor weight over time; however, we did not detect an exponential relationship between time and tumor weight, indicating that tumor growth rate began to decline after their detection. Thus, we did not discuss tumor growth rate in the revised manuscript.

**Figure R4.** Relationship between time since tumor detection and log_10_ tumor weight.

6) *Tumor multiplicity*. Tumor multiplicity (i.e., the number of tumors per animal) is used as a measure of tumor development for experimental models where more than one tumor usually develops, as in the present rat model. As the data we present in Table 3 (“Tumors per rat” columns) were influenced by the difference in the observation period (“Age at autopsy” in the same table), we adjusted tumor multiplicity data according to the observation period and made comparisons among the three strains. As the graphs below illustrate, this analysis suggested the same tendency as detected in the development of first palpable tumors (Fig. 1A and B). Thus, tumor multiplicity does not provide any particular insights.

**Minor comment iv**

iv) Abstract lines 45-46. This is confusingly written. If the incidence in F1 animals is intermediate it cannot be similar to SD. If it were similar to SD then the “sensitivity” genes in SD are dominant over the “resistant” genes in COP.

**Response:** We have modified the sentence as suggested.

Lines 45–48 (Abstract): “Irradiated Copenhagen rats exhibited a very low incidence of mammary cancer. Unexpectedly, this resistance trait did not show dominant inheritance in F_1_ rats ~~but~~; rather, they exhibited intermediate susceptibility levels (i.e., between those of their parent strains) ~~similar to that of Sprague-Dawley rats~~.”

**Minor comment v**

v) Benign mammary tumors? What are these and how were they defined?

**Response:** We have added an explanation of benign tumors to the Abstract.

Lines 49–50 (Abstract): “The susceptibility of irradiated F_1_ rats to the development of benign mammary tumors (i.e., fibroadenoma and adenoma) was also intermediate.”

**Minor comment vi**

vi) Line 80. The “combination” of SD and COP rats …. I think this is not a clear term, it probably refers to studies on F1 hybrids?

**Response:** We have modified the sentence as suggested.

Lines 104–106 (Introduction): “~~The combination of~~ (SD ~~and~~ × COP)F_1_ rats thus provide~~s~~ a good opportunity to investigate the inheritance of susceptibility to radiation-induced mammary carcinogenesis.”

**Minor comments vii and viii**

vii) Line 84-87. The authors need to clarify if the AI on Chr1 is due to gain or loss.

viii) What do the authors mean by saying some of the CNV are allelic imbalances?

**Response:** We understand that these two comments relate to the same part of the text (lines 109–113, Introduction, in the revised manuscript). The text indicated by comment vii describes a previous study from a different laboratory, which did not investigate DNA copy number aberrations. Regarding comment viii, we admit that the original text was not clear about how assessment of allelic imbalance differs from that of copy number aberration.

As we had another comment from Reviewer 2, pointing out that the Introduction of the original manuscript was too technical, we did not choose to explain all of these issues in the Introduction, but simply changed the text “allelic imbalance (AI)” to “genomic changes” and made some clarifications. We have responded to the present comments in other parts of the revised manuscript too, as follows:

Lines 108–121 (Introduction): “Genetic susceptibility to cancer and environmental factors are often linked to specific types of somatic mutations. For example, non-random ~~allelic imbalance (AI; the increase or decrease in one of the two alleles of a gene) is~~ genomic changes ~~observed~~ in chromosome 1 are observed in chemically induced mammary tumors in certain strains, including (WF×COP)F_1_ rats, but not in radiation-induced cancers [18, 19]. As ~~resistant strains are expected to have~~ a potent tumor-suppressor (or oncogenic) gene(s) ~~that~~ is expected to be disrupted (or enhanced) in induced cancers in resistant (or susceptible) strains, non-random changes in tumors strongly suggest the existence of potent tumor-related gene(s). Thus, hybrid ~~animal~~s between strains with different tumor susceptibility offer ideal experimental tools for identifying potentially causative mutations. A~~n~~ successful example is the F_1_ hybrid of susceptible C57BL/6 and resistant C3H mice, which develops radiation-induced thymic lymphoma with ~~loss of heterozygosity (the decrease in one of the two alleles)~~ genomic changes in chromosomal regions spanning critical tumor-suppressor genes [20, 21].”

Lines 197–204 (Materials and Methods): “**Analysis of allelic imbalance (AI) ~~analysis~~.** AI of a simple sequence length polymorphism genomic DNA locus was assessed by comparing the intensities of two DNA bands amplified with the same PCR primer set. Note that, when intensity of one of the two bands (which represent the two alleles at this locus) is stronger than the other, the imbalance can indicate gain of that allele or loss of the other allele, as the PCR assay used is competitive, rather than quantitative. The primer sequences were ~~Genomic DNA was amplified by PCR using primers for single-strand length polymorphism markers~~ obtained from the Rat Genome Database [29] …”

Lines 320–322 (Results): “A previous study on radiation-induced mammary carcinoma of (WF×COP)F_1_ rats indicated that AIs ~~in these tumors~~ occur at very low frequency (4–13%) in these tumors; the study did not relate these AIs to copy number aberrations [18].”

**Minor comment ix**

ix) The text on lines 131-133 is trivial and does not need to be included.

**Response:** We have removed the relevant text as suggested.

Lines 171–175: “Genomic DNA ~~that was used~~ for microarray analysis was extracted ~~from pestled tissues by digestion with proteinase K and ribonuclease A in 0.5% sodium dodecyl sulfate followed by phenol/chloroform extraction and ethanol precipitation~~ as described previously [26].”

**Minor comment x**

x) The criteria were used to select and mark the loci selected in Figure 2 in blue are not given in enough detail and appear to be restricted to the largest chromosomes. For example changes around the Nbl1 locus are only seen in 1 tumor.

**Response:** We have corrected the main body (please see the magenta lines below, which have been added for reviewing purposes only) and the legend of Figure 2. *Nbl1* should be located at chromosome 5q36, rather than 3q34 (where *Ctnnbl1* is located; we mistook *Ctnnbl1* for *Nbl1* in the search system). In addition, the legend in the original manuscript referred to ‘Table 4’ (i.e., Table 6 in the revised manuscript), but this should be ‘Figure 4’ (expression of genes in the affected regions of chromosomes 1, 2, 3 and 5).

Lines 305–309 (legend of Figure 2): “**Fig 2. Copy number aberrations in ~~rat~~ mammary carcinomas of (SD×COP)F_1_ rats.** A, ~~Shown are c~~ Chromosomal losses (green) and gains (red) in 21 tumors. Aberrations observed in the same carcinoma are arranged vertically, whereas those in different carcinomas are side by side. Locations of ~~relevant~~ genes examined in the expression analysis are shown in blue (see Figure ~~Table~~ 4 and text). …”

**Minor comment xi**

xi) The data shown in figure 3 is not clearly described. How was the assignment of gain/loss made, and how many tumors were showing the indicated changes. What is H in the individual boxes signifying?

**Response:** We have improved the clarity of the text, the figure legend, and the figure itself.

Lines 322–343 (Results): “As the SD strain is an outbred population and genetically heterogeneous, we first searched for simple sequence length polymorphism markers that showed heterozygosity in (SD×COP)F_1_ rats and their parental strains, using genomic DNA obtained from normal ear skin. This search identified 42 markers that showed heterozygosity in ~~hosts of 4–21 tumors in the present population of 30~~ tumor-bearing (SD×COP)F_1_ rats (Fig. 3, markers in black letters with asterisks). We found 35 instances of AI across 20 markers in ~~In the genome of~~ mammary carcinoma genomes ~~of~~ from (SD×COP)F_1_ rats~~, these markers were located at 20 sites with AI~~ (Fig. 3, COP and SD). … ~~For~~ At several sites, copy number variations identified using microarrays did not accompany AI, indicating the relatively low sensitivity of AI detection, which is understandable, considering the possibil~~e~~ity for contamination of tumor samples with non-malignant cells (e.g., stromal cells) ~~in the tumor samples~~. The majority of observed AIs ~~were mostly~~ (28/35) were accompanied by microarray-identified copy number loss (Fig. 3, SD and COP on light-grey background), suggesting that these AIs reflected a loss of heterozygosity produced by large deletions. In contrast, AIs accompanying microarray-identified copy number gain were rare (2/35; Fig 3, SD and COP on black background).”

Lines 348–354 (legend): “**Fig. 3.** **Allelic imbalance~~s~~ (AI) sites observed in (SD×COP)F_1_ mammary carcinomas.** Results of the AI analysis along with information from ~~the~~ microarray-based copy number analysis (Fig. 2), indicated in grey scale. Columns indicate individual tumors. Note that copy number variations irrelevant to the indicated markers are not shown. *^a^*Markers with (black with asterisk) or without (grey) heterozygous individuals. *^b^*No data present in the rn4 rat genome assembly; values in parentheses are from the Celera assembly. ‘H’, markers exhibiting homozygosity, where allelic analyses were impossible.”

**II. RESPONSE TO REVIEWER #2**

We wish to express our appreciation to the reviewer for their insightful comments. Below, we have addressed the comments and included the revisions made. Line numbers refer to those in the manuscript with track changes. Changes are indicated by the following rule.

1. Underlined: Added text

2. ~~Strikethrough~~: Deleted previous text

3. Red text: Changes relevant to the present comment

We have had all revised texts reviewed by two native English-speaking professionals at Bioedit (https://www.bioedit.com/).

**Overall remarks**

The manuscript submitted by Nishimura and Co is a study designed to investigate the role of genomic susceptibility in radiation-induced cancer.

The study deals with an important issue after radiotherapy. The experiment was designed and performed well, but the manuscript didn’t report and interpret data well enough.

**Response**: We appreciate the reviewer’s pertinent remarks on the present paper. Please see below our point-by-point responses to each comment.

**Comment 1**

The first issue is that authors need to rearrange (shape) the introduction and discussion in the focus of readers of PLOS ONE. The authors need to start both sections with issues about radiation-induced cancer, epidemiological knowledge, and the topics like genomic susceptibility, individual sensitivity, age and gender in the context of available knowledge about radiation-induced cancer. They can of course argue the lack of suitable in vivo or in vitro model in the field and promote their model. Otherwise, the paper remains at a level of a technical report to introduce an animal model for such a study. If this is the aim of the paper, then they need to negotiate with the editor to submit the paper as a technical report.

**Response:** We have modified the beginning of the Introduction and Discussion sections, as suggested. We kept the additional text in the Discussion as concise as possible to avoid repetition of information stated in the Introduction. We did not choose to submit the present paper as a technical report.

Lines 62–83 (Introduction): “Exposure to ionizing radiation is common in the modern world and can induce various types of DNA damage, including double strand breaks. Some DNA double strand break repair systems are inherently error-prone; therefore, radiation exposure can result in mutations, such as large deletions, translocations, and reversions, and can ultimately disrupt the integrity and/or expression of cancer-related genes [1]. Thus, radiation is a risk factor for cancer development in humans. Epidemiological studies have been conducted on populations exposed to radiation from various sources, including atomic bombs, medical devices, nuclear industry workplaces, contaminated environments, and natural background (see [2] for an example). These studies have clarified that cancer risk increases with radiation dose in a manner compatible with a linear response, without a threshold, at low doses and low dose rates [2]. They also suggest that the response can be modified by individual factors such as age, sex, and lifestyle factors, including cigarette smoking [3]. Genetic variation is another contributor that governs individual susceptibility to cancer. Familial cancer exhibits a high probability of inheritance and constitutes approximately 5% of all cancers [4], and large numbers of genetic polymorphisms are considered to influence cancer risk in a more subtle manner [5]. Although some genetic factors that influence the risk of developing acute tissue reactions after high doses of radiation have been identified, little is known about genetic factors that interact with radiation-induced cancer [3]. Clarification of such interaction would be of benefit when considering radiation use in clinical settings, as well as for the selection of emergency workers and astronauts who will be exposed to relatively high doses of radiation [3].” *(The double-underlined text has been moved from the end of the subsequent paragraph in the Introduction.)*

Lines 388–393 (Discussion): “Epidemiological studies have established that radiation exposure is a risk factor for cancer development in humans, and determining the genetic factors that interact with radiation in this context is vital to understanding individuals’ responses to radiation [3]; however, screening of genetic polymorphisms related to environmental cancer risk in humans generally requires a massive sample size [7]. In this regard, a~~A~~nimal models are useful for studying the role of gene-environment interactions in cancer susceptibility…”

**Comment 2**

Even in a form of technical paper, authors still need to justify the selected criteria such as the age of animals, the dose and the dose rate, the time after exposure.

**Response:** We chose to use the current radiation dose (4 Gy), dose rate (0.5 Gy/min), and age at irradiation (7 weeks) based on data from previous studies (refs. 17, 23, and 24). These conditions have been proven to induce significant mammary carcinogenesis in SD rats. Mammary carcinogenesis exhibits a dose-dependent increase in SD rats up to at least 4 Gy; lowering the radiation dose rate significantly reduces incidence of both carcinoma and benign tumors (ref. 17). Our previous experiments indicated that irradiation of 3-week-old rats at 2–4 Gy results in attenuated mammary carcinogenesis, compared with use of 7-week-old rats (refs. 23 and 24). We have thus added a sentence regarding the choice of exposure conditions. Furthermore, because radiation exposure conditions can be variable and are a matter of intense investigation, we have added a brief discussion on this point to the revised manuscript (please see our response to comment 3).

Regarding the time after exposure, animals were followed for their entire life span, during which weekly palpation was conducted to monitor tumor development. Thus, a specific time after exposure was not selected in the current experiment.

Lines 143–147 (Materials and Methods): “Briefly, 7-week-old female rats were subjected to whole-body γ-irradiation~~ed~~ (^137^Cs, 4 Gy, 0.5 Gy/min) and then palpated weekly for the remainder of their lifetime to detect tumors. The dose and dose rate of radiation and the age at irradiation were chosen as they are established to induce maximal mammary carcinogenesis, based on our previous studies [17, 23, 24].”

**Comment 3**

Is there any available data that a higher or lower dose and a long(er) time after exposure can affect the gene profile analysed in the present study differently?

**Response:** We analyzed the correlation between age at tumor detection (which is equivalent to the time after exposure, as all rats were exposed at the same age) and genetic copy number and gene expression changes. We found positive correlations between age at detection and i) the number of breast cancer-related genes involved in regions of copy number loss (Figure R5A, *P* < 0.01) and ii) the number of chromosomes with large deletions (spanning > 80% of the chromosome, Figure R5B, *P* < 0.01), implying that some tumors develop via accumulation of large chromosomal deletions, which requires time, whereas those that develop early may be a consequence of other mechanisms, such as point mutations, inversions, and translocations. We did not detect any significant correlation with age at autopsy or the time between tumor detection and autopsy. Thus, time after exposure influences the genetic changes in tumors.

A

Genes involved in loss

| Age at detection (weeks) | TOTAL (LOSS) |
| --- | --- |
| 16 | 0 |
| 18 | 0 |
| 42 | 14 |
| 42 | 1 |
| 22 | 0 |
| 62 | 2 |
| 41 | 4 |
| 76 | 6 |
| 57 | 11 |
| 50 | 2 |
| 62 | 1 |
| 78 | 47 |
| 61 | 3 |
| 88 | 0 |
| 69 | 7 |
| 90 | 7 |
| 77 | 4 |
| 68 | 5 |
| 93 | 35 |
| 92 | 9 |
| 104 | 14 |

B

No. of chromosomes affected

| Age at detection (weeks) | Total-Loss (>80%) |
| --- | --- |
| 16 | 0 |
| 18 | 0 |
| 42 | 2 |
| 42 | 0 |
| 22 | 0 |
| 62 | 0 |
| 41 | 0 |
| 76 | 1 |
| 57 | 2 |
| 50 | 0 |
| 62 | 0 |
| 78 | 9 |
| 61 | 0 |
| 88 | 0 |
| 69 | 1 |
| 90 | 1 |
| 77 | 0 |
| 68 | 0 |
| 93 | 6 |
| 92 | 1 |
| 104 | 3 |

**Figure R5.** Correlation between age at tumor detection and genomic changes in tumors. A, Number of breast cancer-related genes affected by copy number loss. B, Number of chromosomes with > 80% copy number loss.

We have added these results as Fig. 2B and 2C in the revised manuscript, along with relevant methods and discussion.

Lines 227–228 (Materials and Methods): “The significance of correlations was assessed by Spearman’s rank correlation test.”

Lines 295–300 (Results): “Copy number gains were relatively rare (Fig. 2A). We identified many genes relevant to human breast cancer [27] in the chromosomal regions exhibiting copy number changes (Table 46). Interestingly, the number of genes affected by copy number loss was positively correlated with age at tumor detection (Fig. 2B, *P* < 0.01), which could be explained by correlation with the number of chromosomes with large deletions spanning > 80% of the chromosome (Fig. 2C, *P* < 0.01).”

Lines 300–307 (legend of Fig. 2): “**Fig. 2. Copy number aberrations in rat mammary carcinomas from ~~of~~ (SD×COP)F_1_ rats.** A, ~~Shown are~~ Chromosomal losses (green) and gains (red) in 21 tumors. Aberrations observed in the same carcinoma are arranged vertically, whereas those in different carcinomas are side by side. Locations of ~~relevant~~ genes examined in the expression analysis are shown in blue (see Figure ~~Table~~ 4 and text). B and C. Correlation between age at tumor detection and genomic changes in tumors. B, Number of breast cancer-related genes affected by copy number loss. C, Number of chromosomes with > 80% copy number loss.”

Lines 473–477 (Discussion): “Our present analysis indicated a positive correlation between large deletions and age at tumor detection, implying another benefit of the long observation period. This observation suggests that some tumors develop via accumulation of large chromosomal deletions which requires time, whereas those that develop early may use other mechanisms, such as point mutations, inversions, and translocations.”

The radiation dose-dependence of the genetic changes in mammary tumors remains open to future investigation because the present experiment used only a single radiation dose (4 Gy) for (SD×COP)F_1_ rats. Besides the radiation dose, multiple factors are potentially relevant to modification of the strain difference observed herein. We have added this as a limitation and a perspective for future study in the Discussion section.

Lines 507–513 (Discussion): “The present study has the following limitations. First, … Second, our results may have been affected by variation in exposure conditions, such as radiation dose, dose rate, fractionation regimen, radiation type, age at time of exposure, hormonal conditions at time of exposure, and whether the whole or partial body was irradiated. This point remains open for future study.”

**Comment 4**

The authors managed to provide evidence that distinguished their model from previous studies, they need to highlight those such as multiple copies no variation and try to translate it to a human scenario. As mentioned before, the obtained data need to be discussed in the context of radiation-induced cancer.

**Response:** As suggested, we have added following discussion to the revised manuscript.

Lines 495–506 (Discussion): “In humans, sporadic breast cancers generally exhibit multiple DNA copy number aberrations, consistent with the present animal model. By contrast, there is little evidence of genetic aberrations in radiation-induced breast cancer in humans. In the present study, the regions showing copy number aberrations in tumors from (SD×COP)F_1_ rats did not necessarily correspond to those previously reported in SD rats [33]. This strain difference suggests that copy number aberrations in radiation-induced breast cancer are subject to the influence of genetic background. In fact, in a study of human breast cancers that developed as second primary cancers after radiotherapy (*n* = 3 tumors), no common deletions or inversions that could be causative were reported; rather, multiple deletions and inversions of non-coding regions were reported [46]. Larger investigations are thus warranted to clarify the common genetic changes detected in radiation-induced human cancers.”

**III. MISCELLANEOUS CHANGES**

Line 6: “Masaru Takabatake^2^” (*We corrected a discrepancy between the author information on the editorial system and in the manuscript, where information on the former was correct.)*

Lines 446 and 448: “*Map~~k~~3k1*”

Lines 449–451: “Analysis in humans indicated that *MAP3K1* is the gene in the corresponding region (human chromosome 5q11.2 ~~of rat~~) with the greatest influence on risk of breast cancer development [39].”
